# Reminiscences of the Intelligence of Animals

**Published:** 1895-11

**Authors:** Isaiah Michener

**Affiliations:** Carversville, Bucks Co., Pa.


### CORRESPONDENCE.

### REMINISCENCES OF THE INTELLIGENCE OF ANIMALS.

EDITOR JOURNAL OF COMPARATIVE MEDICINE.

DEAR SIR: Believing that our domestic animals have more knowledge than they have been credited with, I propose giving some of my experience in that line.

TEACHING A DOG TO HUNT. When I was sixteen years old a neighbor boy and I each obtained a half-bred hound dog for hunting rabbits, musks, skunks, opossums, and raccoons, which were numerous enough in those days to afford a great amount of sport for boys to hunt them.

As soon as our dogs were old enough we began training them to hunt raccoons. For that purpose we obtained a 'coon, which we would lead out in the evenings, occasionally run him up a tree ten feet high, then pull him down and carry him fifteen or twenty yards before letting him touch the ground. The dogs never seemed to lose the scent where the 'coon was carried above ground, nor they never stopped to bark at the tree where the 'coon was run up and pulled down. When our best dog would find the tree that the 'coon was on, he would make a circuit for, perhaps, twenty yards from the tree, all around it, then take the back track and run it a short distance, then stop short and come back as fast as he could run toward the tree, as if he would knock his brains out against it; but instead of that, he would run up the tree as high as ten feet, then stiffen his legs and throw himself back on to his feet, and bark sharply several times. Then he was done, and by no means could he be induced to hunt any more, and when urged to do so, he would show by his countenance utter disgust.

Now if young veterinarians would show as much caution and good judgment in diagnosing diseases as that dog did in treeing 'coons, there would be fewer mistakes made than there is at the present day. Can any man show more caution and intelligence in coming to right conclusions than that dog did?

AN INTELLIGENT HORSE. I raised a half-bred Canadian horse, named "Bill," that soon learned to untie any knot in a rope or strap, so that it was out of the question to tie him in that way, so I obtained a chain with a spring snap, like a trace-chain, with a T to the other and, and ran it through two holes, the last hole put where he could not get at it. Now I thought I had outwitted him; but in less than one week he learned to unloose the spring under his jaw on the top of the manger by pressing his jaw down on it, at the same time giving his head a rotary motion and pull back and snap the spring and be loose; then turn to the door behind him, lift the hook out of the staple, push the door open, and walk out into the yard; then go to the entry door, that was fastened with a wooden latch, catch it with his teeth and lift it up, and pull the door back and walk into the entry; go direct to the feed-chest, which had a lid on it three feet square and heavy. He would take hold of the edge and lift it up; then try it again, with the same result; then he would get mad, seize it and throw it back with great force against the wall; then eat all the food he wanted, walk out into the barnyard, and amuse himself walking about; but woe be to any stranger that would venture into the yard to drive him into the stable, for he would go for them with all the vengeance there was in him.

On one occasion when masons and carpenters were at work for me he loosened his chain and walked out. The hired man tried to get him in the stable, but the horse turned upon him and drove him out of the yard. Then the mechanics, three of them, undertook the job, and would have been trampled to death if they hadn't made a hasty retreat. At that critical moment I arrived home. The men were all looking over the fence at the horse quietly eating in the yard. As I entered the yard the horse turned toward me with his ears lying back on his head, ready to jump on me. I yelled out, "Bill! What do you mean?" He stopped as quick as if shot. I walked up to him and laid my arm on his neck and said, "Bill, walk with me into the stable," which he did as quietly as any horse could.

I write these facts in regard to the intelligence of horses and dogs, thinking that they might be read to students to teach them to study more carefully the disposition of our domestic animals, and thereby govern them intelligently and kindly rather than with brute force.

WHY "MAZEPPA" SO PERFORMS. I intended to give my views on the horse "Mazeppa's" performance, but have missed or lost the number that I read the account in, so I cannot review it as I would like to.

Animals that perform as Mazeppa does are brought under psychological influence by him who manages them; they are the medium through which the psychologist performs the work, whilst the animal is entirely ignorant of and knows nothing about what he is doing, the same as a person hypnotized will do whatever the hypnotizer orders him to do without having any idea of what he is doing. After the influence is taken off of the hypnotic patient, if he is questioned in regard to what he had said or done he will tell you that he is not aware that he said or had done anything. We had at one of the Doylestown fairs what was called a "learned pig" on exhibition, which the managers were invited in the tent to see. On entering the place I at once remarked, "That hog is not in a normal condition." He was moping about with his head down moaning all the time. I called the attention of a doctor to it, but he could not see anything wrong. I told him that his practice didn't cultivate the perceptive faculties like mine did, which was the reason he could not see it (excuse the digression).

I observed that the manager would frequently drop some corn from his pocket, and at the same time pass his leg down over the hog's head and face, and then, for instance, tell him to hunt out the cards that would spell out the name of the President of the United States. The hog would go to the cards and soon pick out what would spell Lincoln, and so on, whatever he was directed to do he would perform. The man performs these feats through the medium of a hypnotized hog's brain.

This theory may be tested. Request the man to order Mazeppa to do a thing that he doesn't know how to do himself; for instance, how many bones are there in a horse's knee. See if Mazeppa will stamp them out, or any other proposition that you know the man would not understand, and he is able to answer. I think you will soon find Mazeppa stuck fast, stalled.

ISAIAH MICHENER, V.S.

CARVERSVILLE, BUCKS CO., PA., August 14, 1895.

